# Comorbidity-Driven Inflammation in HFpEF: *Immune Profiling and Therapeutic Targets*

**DOI:** 10.1007/s11897-026-00745-0

**Published:** 2026-03-13

**Authors:** Ellaline Cami, Eline Verghote, Emily Pecetto, Arantxa González, Ebba Brakenhielm, Elizabeth AV Jones, Vanessa Van Empel

**Affiliations:** 1 Center for Molecular and Vascular Biology, KU Leuven, Herestraat 49, bus 911, Leuven, 3000 Belgium; 2 Department of Cardiology, Cardiovascular Research Institute (CARIM), Maastricht University Medical Centre, Universiteitssingel 50, Maastricht, 6229 ER Netherlands; 3 Department of Physiology, Cardiovascular Research Institute (CARIM), Maastricht University Medical Centre, Maastricht University, Universiteitssingel 50, Maastricht, 6229 ER Netherlands; 4 Program of Cardiovascular Diseases, Centro de Investigación Médica Aplicada (CIMA), Department of Cardiology and Cardiac Surgery, Department of Pathology, Anatomy and Physiology, Universidad de Navarra, Clínica Universidad de Navarra, Universidad de Navarra, IdiSNA, Avd. Pío XII 55, Pamplona, 31008 Spain; 5 Centro de Investigación Biomédica en Red Enfermedaded Cardiovasculares (CIBERCV), Instituto de Salud Carlos III, Av. Monforte de Lemos 3-5, Madrid, 28029 Spain; 6 University Rouen Normandie, INSERM EnVI, UMR1096, Rouen, F-76000 France

**Keywords:** (max 6): Cytokines, HFpEF, Comorbidities, Inflammation, Heart failure, Immune profiling

## Abstract

**Purpose of Review:**

Chronic low-grade inflammation underlies the heterogenous pathophysiology of heart failure with preserved ejection fraction (HFpEF) influenced by patient comorbidities. This review summarizes circulating inflammatory mediators in HFpEF, explores how comorbidities shape distinct immune signatures, and discusses current and emerging treatment strategies.

**Recent Findings:**

Comorbidities, including hypertension, obesity, type 2 diabetes, and chronic kidney disease, drive distinct immune profiles in HFpEF through dysregulated cytokine signaling. Circulating mediators, including IL-6, IL-1β, TNF-α, soluble ST2, and CRP, reflect this comorbidity-driven immune activation and predict adverse outcomes. Current therapies, e.g. SGLT2 inhibitors, mineralocorticoid receptor antagonists, and angiotensin receptor-neprilysin inhibitors, display anti-inflammatory effects but benefit only specific subgroups. Emerging inflammation-targeted strategies, including anti-IL-6 or anti-IL-1β, NLRP3 inflammasome modulation and myeloperoxidase inhibition, are under clinical investigation.

**Summary:**

Linking immune profiles to HFpEF phenogroups may enable precision medicine by refining risk stratification and tailoring therapies, moving beyond the current one-size-fits-all approach.

## Introduction

Heart Failure (HF) with preserved ejection fraction (HFpEF) is a heterogenous syndrome associated with multiple comorbidities, including hypertension (HTN), obesity [1, 2], type 2 diabetes (T2D) [3], atrial fibrillation (AF), and chronic kidney disease (CKD) [4]. Inflammation may play an important role in the development of HFpEF, often presenting as chronic, low-grade inflammation [5]. Clinical studies highlight different circulating pro- and anti-inflammatory markers in HFpEF, including increased interleukin-6 (IL-6) and tumor necrotic factor (TNF)-α levels [6, 7]. Though many HFpEF-associated comorbidities are linked to chronic systemic inflammation, they may activate immune responses through distinct mechanisms.

While inflammation is a shared finding in most HFpEF patients, the inflammatory profiles may differ based on factors such as stage of the disease, but also age, gender, and the cumulative load of comorbidities. This phenotypic heterogeneity in HFpEF is becoming increasingly recognized, putting to question the assumption that all patients will benefit from similar treatments. Obesity, kidney dysfunction, HTN, T2D, gender and age all impact the immune system, including cytokine production. Cardiac fibrosis, a hallmark of HF including HFpEF, is largely driven by inflammatory mediators released in the heart by infiltrating immune cells, including monocytes and macrophages, T cells, B cells, neutrophils, mast cells, and dendritic cells [8–11]. Promisingly, clinical trials have indicated that anti-cytokine therapies, and other immune-modulatory treatments, may limit not only cardiac inflammation and dysfunction, but also cardiac remodeling including fibrosis [12, 13]. Thus, inflammation has emerged as a major therapeutic target in many cardiovascular diseases, including HFpEF.

Cytokines are key actors not only regulating immune cell activation, but also orchestrating, via chemokines, the recruitment of immune cell subpopulations to the heart. The specific comorbidities driving HFpEF development may contribute to define the inflammatory environment in the heart. Consequently, as HFpEF patients also may be heterogenous in terms of circulating or cardiac inflammatory mediators, there is a need for new better tailored treatment approaches adapted to patient-specific immune profiles. Circulating inflammatory biomarkers may provide useful clues to identify: (1) subgroups of HFpEF patients with the most pronounced immune activation; and (2) potential treatment targets specific for these patients.

### Inflammation and the Immune Profiles in Patients With HFpEF

Metabolic stress, as a result of the comorbidities seen in HFpEF, induces low grade inflammation, impacting the nitric oxide (NO) availability via the presence of pro-inflammatory cytokines and the increase of reactive oxygen species (ROS) [6]. In physiological conditions, NO released by endothelial cells exerts a fundamental vasodilating effect necessary for a proper microvascular function. Impaired NO production, contributes to microvascular dysfunction seen within HFpEF. Additionally, endothelial cells activation, triggered by increased cytokines levels, results in activation of adhesion molecules. This promotes infiltration of circulating leukocytes and myocardial inflammation, resulting in increased myocardial fibrosis [13]. Monocytes will infiltrate the myocardium, differentiate to macrophages and produce profibrotic transforming growth factor beta (TGF-β). TGF-β will activate fibroblasts to differentiate into myofibroblasts that will deposit collagen, inducing fibrotic tissue [13, 14].

The lack of NO dysregulates the NOS/NO-sGC-cGMP-PKG pathway in cardiomyocytes, leading to low levels of protein kinase G (PKG) [6, 13]. By phosphorylating titin, PKG contributes to the elasticity of cardiomyocytes [15, 16]. Low levels of PKG result in loss of phosphorylation of titin, with consequent impaired elasticity of the cardiomyocytes, adding to stiffening of the heart [17] and resulting in cardiomyocyte hypertrophy [14, 15].

**Fig. 1:**
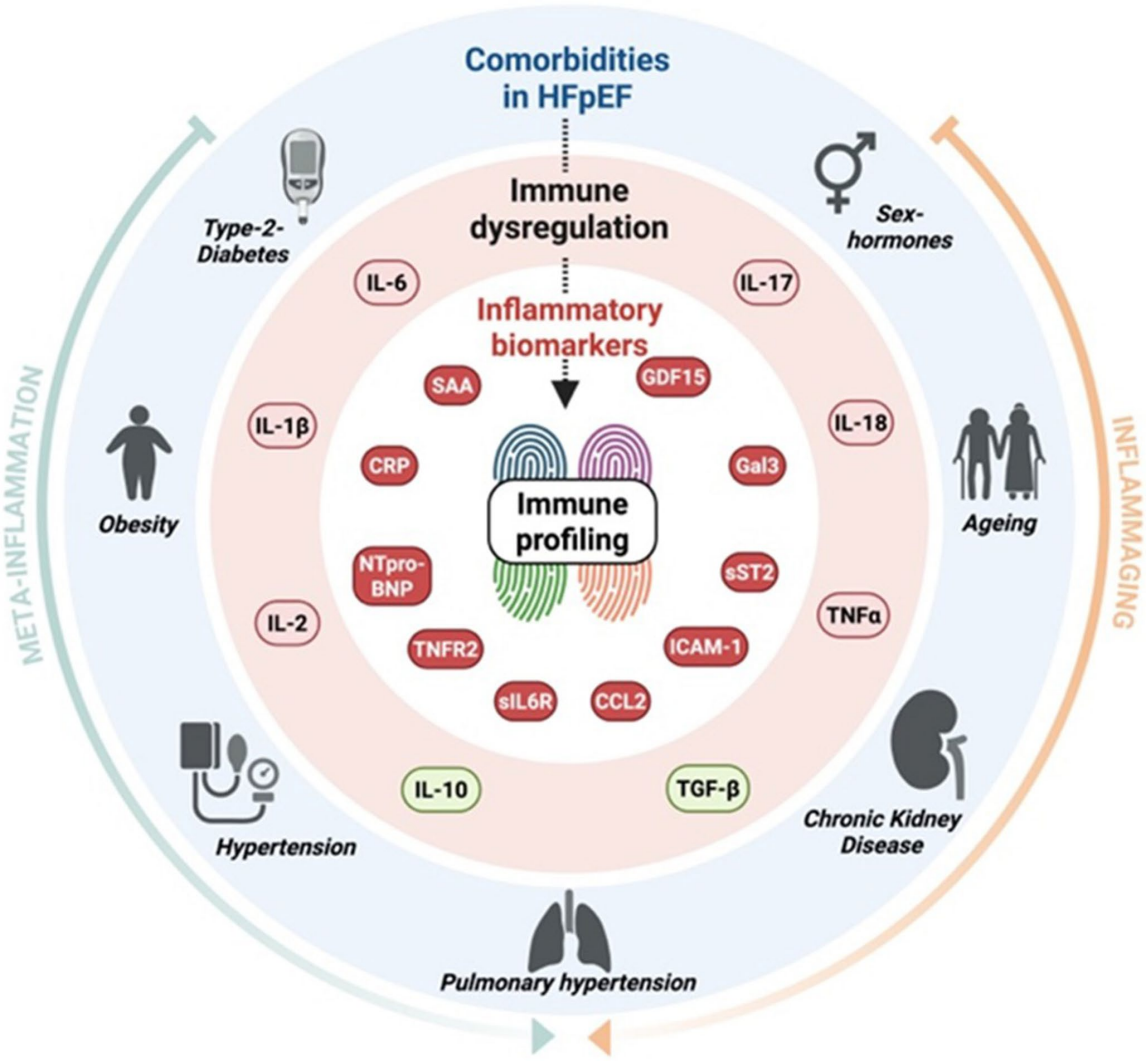
Comorbidity-driven inflammation in HFpEF. Multiple comorbidities, including T2D, obesity, HTN, PH, CKD, but also age and hormonal status, may promote immune dysregulation with altered cytokine signaling (e.g. TNF-α, IL-6, TGF-β), resulting in elevated inflammatory biomarkers (e.g. CRP and NT-proBNP) and distinct circulating immune profiles. Abbreviations: T2D – type 2 diabetes mellitus, HTN – hypertension, PH – pulmonary hypertension, CKD – chronic kidney disease, TNF-α – tumor necrosis factor, IL – interleukin, TGF – transforming growth factor, sIL6R – soluble IL-6 receptor, GDF15 – growth differentiation factor 15, CRP – C-reactive protein, Gal3 – galectin-3, sST2 – soluble suppression of tumorigenicity-2, SAA - serum Amyloid A, ICAM-1 - intercellular adhesion molecule-1, CCL2 - C-C motif chemokine ligand 2, NTproBNP - N-terminal pro–B-type natriuretic peptide, TNFR2 – tumor necrosis factor receptor-2. Created in https://BioRender.com

Though we have an increased understanding of how chronic inflammation develops, the specific role of key immune mediators are still not fully defined. As such, we focus on here on cytokines known to be associated with HFpEF, with special focus on their clinical prognostic values and associations with specific co-morbidities (Fig. 1).

### IL-6

**Fig. 2:**
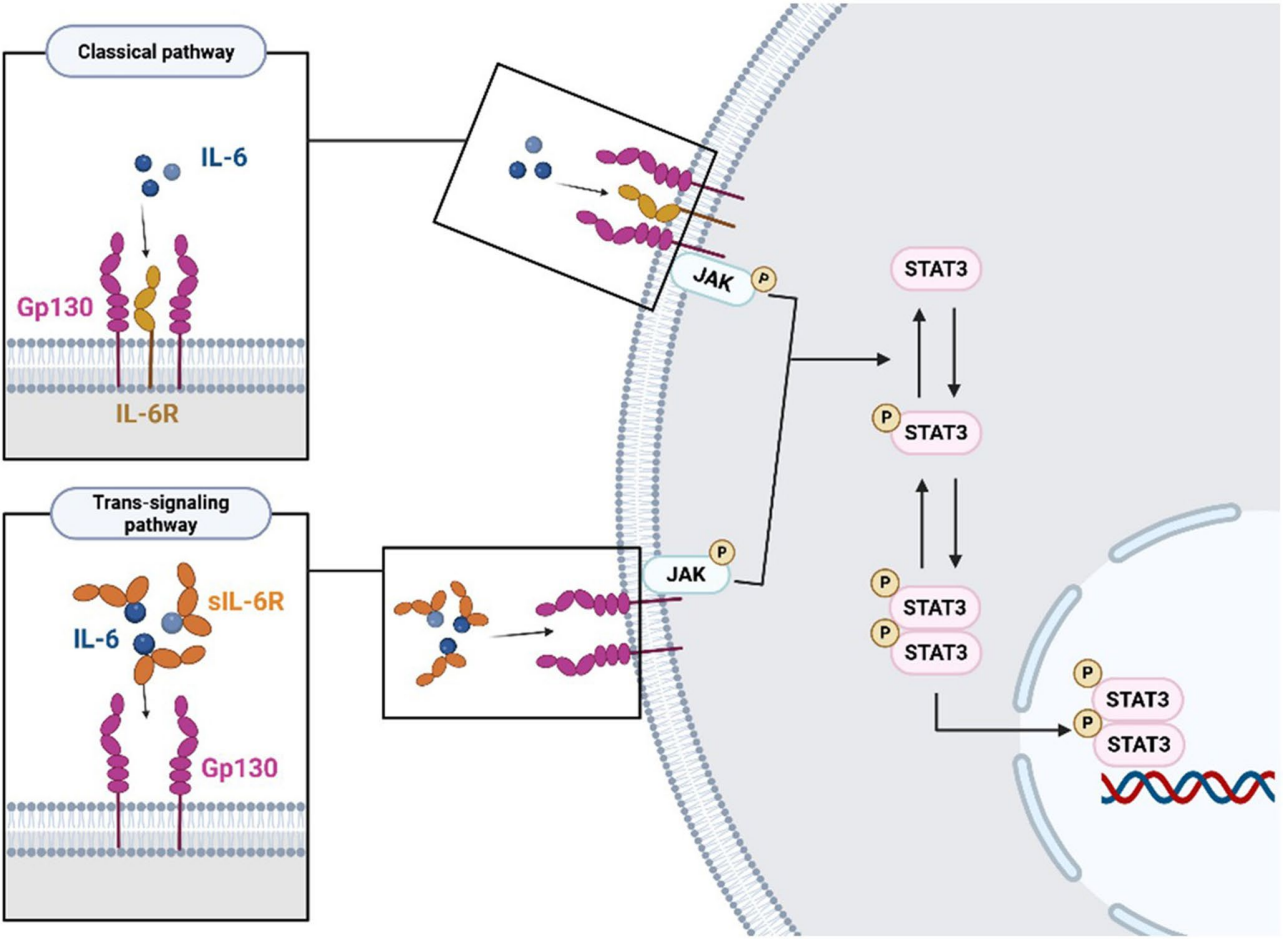
Dual signaling pathways mediated by IL-6: classical and trans-signaling mechanisms. IL-6 can signal through two distinct mechanisms. In the classical pathway, IL-6 binds to membrane-bound IL-6R and recruits gp130, leading to JAK/STAT3 activation and predominantly anti-inflammatory effects. In the trans-signaling pathway, IL-6 interacts with soluble IL-6R (sIL-6R) to form a complex that associates with gp130 on target cells, promoting pro-inflammatory JAK/STAT3 signaling. Abbreviations: JAK – janus kinase, STAT – signal transducer and activator of transcription, IL-6 – interleukin-6, sIL-6R – soluble interleukin-6 receptor. Created in https://BioRender.com

IL-6 is a cytokine that has both pro- and anti-inflammatory properties, depending on the context [18]. It is a small polypeptide with pleiotropic expression (including B- and T-lymphocytes, dendritic cells, fibroblasts, endothelial cells, macrophages, monocytes, and adipocytes) [19, 20]. For example, in obesity, the expansion of adipose tissue is correlated with higher IL-6 plasma levels [19]. The expression of IL-6 is induced by other pro-inflammatory cytokines (e.g. IL-1β and TNF-α), or by cell recognition of damage-associated molecular patterns (DAMPs) or pathogen-associated molecular patterns (PAMPs) by toll-like receptor (TLR). IL-6 binds its transmembrane receptor, IL-6R (expressed notably by immune cells and hepatocytes), which signals in complex with a co-receptor, gp130 (expressed by most cell types). Of note, IL6R is produced and released to the circulation in a soluble form (sIL6R), which acts as a transporter of plasma IL-6, thus extending the cytokine’s half-life. Moreover, the circulating IL-6/sIL6R complex can engage gp130 in the membrane of target cells with low or no IL6R expression, through a mechanism of non-canonical trans-activation signaling [20]. Whereas classical IL6R signaling has anti-inflammatory effects, the IL-6/sIL6R trans-signaling is pro-inflammatory [21]. However, both canonical and non-canonical IL-6 signaling leads to JAK/STAT pathway activation [22] (Fig. 2). The downstream gene targets also differ depending on cell type. For example, upregulation of the acute-phase reactant C-reactive protein (CRP, *see below*) is a major hepatic target of IL-6 [23]. IL-6 also induces the expression of other cytokines, notably *via* activation and polarization of specific T helper (Th) cells (e.g. IL-10 by Th17 cells [24]). In B cells, IL-6 is an essential factor for their terminal differentiation and immunoglobulin (Ig) production [25]. In adipose tissue, IL-6 trans-signaling leads to recruitment of macrophages [26]. Additionally, IL-6 activates vascular endothelium, mainly through non-canonical pathway, leading to increased vascular permeability and enhanced leukocyte transmigration [27, 28]. IL-6 has also been implicated directly in cardiomyocyte hypertrophy, with cardiomyocyte-specific overexpression of both IL-6 and IL6R found to induce left ventricular (LV) hypertrophy in mice [29]. In addition, it also contributes to cardiac fibrosis through direct effects on fibroblasts [30, 31].

In the clinic, circulating IL-6 levels have been reported to be higher in patients with HFpEF compared to those with HF with reduced ejection fraction (HFrEF) [32], and have been associated with T2D [33]. Importantly, IL-6 has prognostic value in HFpEF [34], with elevated levels associated with higher HFpEF incidence [35], but also with poor outcome, including increased risk of death or HF-related hospitalization (DHFA) [36, 37]. Consequently, there are ongoing clinical trials to target IL-6 and its receptors in HFpEF (*see below*).

### IL-1β

**Fig. 3:**
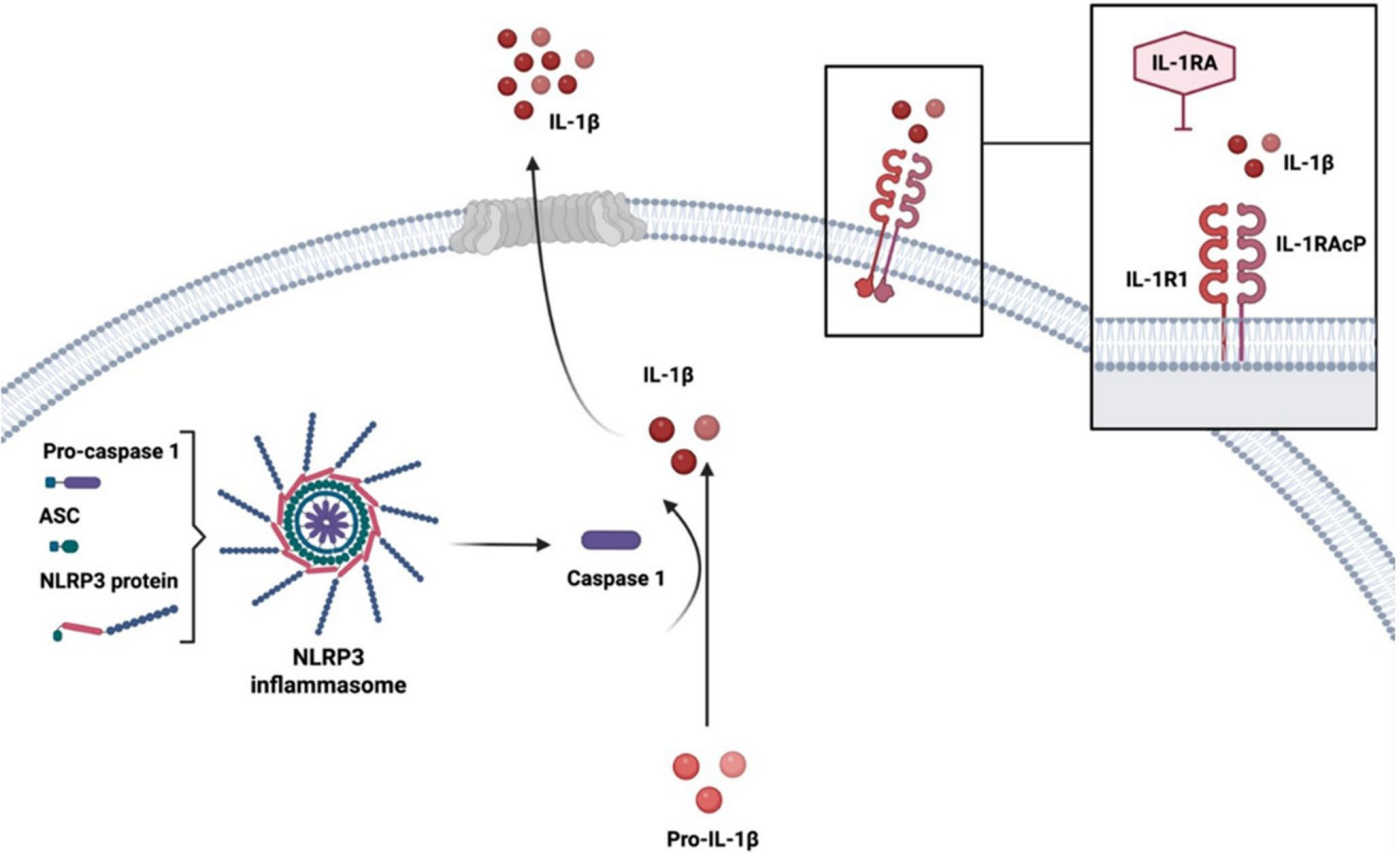
IL-1β activation and receptor signaling. Upon activation, the NLRP3 inflammasome recruits and activates caspase-1, which cleaves pro-IL-1β into its mature, bioactive form that is subsequently secreted. Extracellular IL-1β binds to IL-1R1 and its co-receptor IL-1RAcP to trigger downstream inflammatory signaling, a process antagonized by IL-1 receptor antagonist (IL-1RA). Abbreviation: ASC – Adaptor protein apoptosis-associated Speck-like protein containing a Caspase-recruitment domain, IL-1β – interleukin-1β, IL-1R1 – interleukin-1 receptor type 1, interleukine-1 receptor accessory protein. Created in https://BioRender.com

IL-1β is a pleiotropic pro-inflammatory cytokine, essential notably for innate immune responses [38], expressed at high levels by neutrophils, monocytes, and pro-inflammatory macrophages. It is produced in a precursor form (pro-IL-1β), which is activated by caspase-1-mediated cleavage. In turn, caspase-1 is activated by the NLRP3 inflammasome (*see below*). IL-1β signals via its main receptor IL1R1, with a natural antagonist, IL-1RA, opposing signaling. IL-1β is the prototypical member of the IL-1 cytokine family, which includes 10 additional members, e.g. IL-1α, IL-18, and IL-33. This family shares some signaling receptor components, such as IL-1R accessory protein (IL-1RAcP) (Fig. 3).

 In vivo, IL-1β has negative inotropic effects in the heart [39], whereas in vitro it stimulates the growth of cardiomyocytes [40]. In mice exposed to high fat diet, inhibition of IL-1β improves diastolic dysfunction [41]. In IL-1β knock-out mice submitted to a HFpEF two-hit protocol (L-NAME and high fat diet), the development of fibrosis was prevented [42]. In HFpEF patients, elevated circulating IL-1β levels predicted rehospitalization [43]. Several recent clinical trials have investigated the benefits of blocking IL-1β in HF [38], including HFpEF, e.g. the D-Heart2 trial (*see below*) [44].

HFpEF is a frequent cause of pulmonary hypertension (PH), where IL-1β also appears to play a key pathophysiological role. Indeed, IL-1β was upregulated in pulmonary macrophages in a mouse PH-HFpEF model [45, 46]. Levels were also increased in venous blood, pulmonary arterial blood, and pulmonary capillary wedge blood in HFpEF patients with PH, as compared to non-PH controls. Promisingly, inhibition of IL-1β sufficed to reduce PH development in an experimental murine HFpEF model [45].

### Other Interleukin Family Members

Suppression of tumorigenicity-2 (ST2) is an IL-1 family transmembrane receptor expressed in lymphocytes (e.g. T regulatory cells) but also in cardiomyocytes. IL-33 is the main ligand for ST2, through which it promotes cardiomyocyte survival following ischemic stress [47]. However, both clinical and experimental studies have demonstrated that myocardial injury leads to release of a soluble isoform of the receptor (sST2), functioning as a decoy. In chronic HF patients, increased circulating sST2 levels are associated with worse outcome [48]. Similarly, in HFpEF patients, elevated sST2 levels has been reported [6, 8, 35, 49], and found to be associated with a greater DHFA risk [49].

Other interleukins have been proposed to be involved in HFpEF pathophysiology. For example, IL-2 is a potential biomarker of new-onset HFpEF [50]. Furthermore, increased cardiac expression of IL-10, an anti-inflammatory but also pro-fibrotic cytokine, has been found in the SAUNA (Salt + Unilateral Nephrectomy + Aldosterone) mouse HFpEF model [11]. In the clinic, elevated IL-10 and IL-17 have both been associated with increased risk of HFpEF hospital re-admission [43].

### TNF-α

TNF-α is an inflammatory mediator that has been extensively studied in HFrEF [51]. It binds to the TNF receptors 1 (TNFR1), and 2 (TNFR2), or their soluble isoforms. TNFR1 is present in almost every cell, whereas TNFR2 is expressed solely in immune cells [52]. In the circulation, TNF-α is found either unbound or bound to its soluble receptors. TNF-α differentially impacts different cell types within the heart: it has a negative inotropic effect in cardiomyocytes; it stimulates production of pro-inflammatory cytokines in macrophages; and it impacts cardiac fibroblast-regulated extracellular matrix (ECM) turnover [51]. In the vascular endothelium, it induces (micro)-vascular dysfunction and upregulation of immune cell-adhesion molecules, including VCAM-1 [53].

Circulating TNF-α levels are elevated in HFpEF patients compared to healthy controls [54], and predicts poor outcome [37, 51]. It also has prognostic value for HFpEF incidence [35] and is associated with more severe cardiac hypertrophy [55]. Putko et al. found that higher TNF-α levels are associated with low glomerular filtration rates (eGFR), HTN, smoking status, and history of AF in HF patients [54]. In addition, TNF receptors have been found to be dysregulated in HFpEF. In particular, plasma TNFR2 were higher in HFpEF as compared to both healthy controls and HFrEF patients [54]. Elevated TNFR2 correlated with worse diastolic dysfunction, as well as with advanced age, low eGFR, T2D, and history of AF. TNFR2, together with TNFR1, was predictive of increased E/e’, and advanced NYHA functional class. Indeed, emerging evidence suggests that soluble TNFR1 may serve as a mediator between comorbidities and cardiac remodeling and dysfunction, contributing to poor outcome in HFpEF [8, 56, 57], further supporting the idea that inflammation may be comorbidity-driven.

### Inflammasome

The NLRP3 inflammasome is a protein complex consisting of NLRP3, the Adaptor protein apoptosis-associated Speck-like protein containing a Caspase-recruitment domain (ASC), and caspase-1. It is activated by serine-threonine kinase NIMA-related kinase 7 (NEK7) in response to infection or tissue damage [58]. As mentioned above, caspase-1 activates IL-1β, and the inflammasome is necessary for activation of pro-caspase-1. Mice deficient in NLRP3 inflammasome components were resistant to metabolic syndrome in a model of diet-induced obesity, as well as displaying reduced obesity-induced LV-concentric remodeling and cardiac dysfunction [59]. The NLRP3 inflammasome is being targeted in different clinical trials for HF, mainly HFrEF [60].

### CRP

CRP is rapidly produced in the liver in response to systemic inflammation, particularly NLRP3 inflammasome activation, and CRP plasma levels loosely correlate with inflammatory status [61]. CRP activates the complement pathway, essential for opsonization of pathogens.

Another function of CRP is to stimulate the release of pro-inflammatory cytokines, via binding to Fc receptors [62]. Although elevated plasma CRP levels have been found in HFrEF [63], levels may be further increased in HFpEF [64]. Indeed, CRP is not only predictive of HFpEF [7, 65] but also correlates with increased risk of adverse cardiovascular events and all-cause mortality [7, 66]. Intriguingly, CRP levels were found to increase progressively in HFpEF with the number of comorbidities [66]. Among different risk factors, high CRP levels were linked to obesity and AF. However, 40% of HFpEF patients did not display elevated CRP.

### Immune Profiles Linked with Comorbidities in HFpEF

**Fig. 4:**
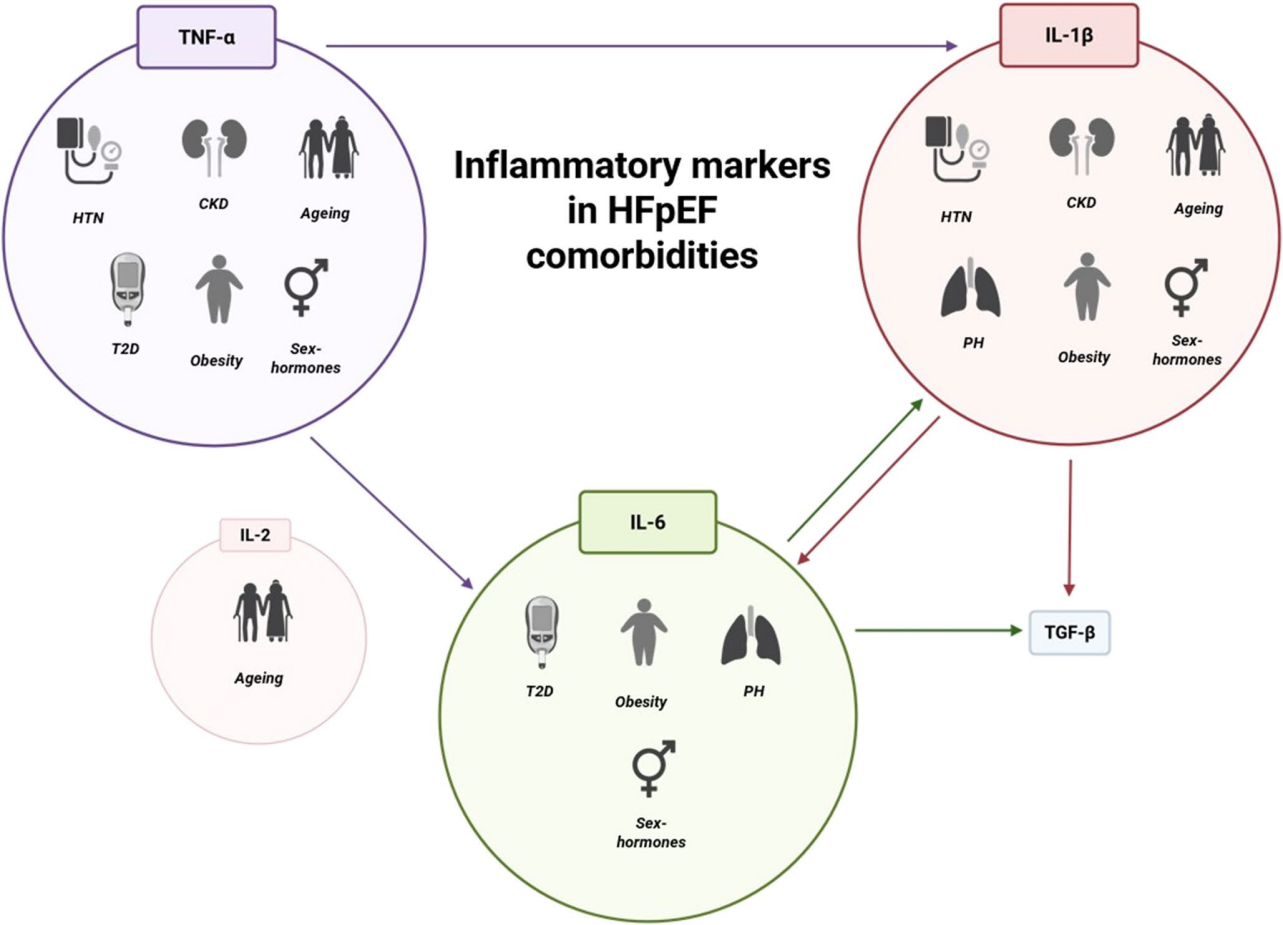
Schematic representation of key inflammatory mediators to major HFpEF comorbidities. TNF-α, IL-1β, IL-6, IL-2 and TGF-β are linked to HTN, obesity, T2D, PH, CKD, ageing, and sex-related factors. Abbreviations: T2D – type 2 diabetes mellitus, HTN – hypertension, PH – pulmonary hypertension, CKD – chronic kidney disease, TNF-α – tumor necrosis factor, IL – interleukin, TGF – transforming growth factor. Created in https://BioRender.com

Different comorbidities associated with HFpEF may induce distinct immune profiles. It has become apparent that not all HFpEF patients have similarly dire prognosis, and those with several comorbidities have the poorest outcomes. Consequently, we and others have proposed clinical-parameter-based phenotyping strategies to better profile cardiovascular risk in HFpEF patients and identify phenogroups with high-risk scores and poor prognosis [67–70]. In the following section, we will review the links between inflammation and individual HFpEF comorbidities (Fig. 4). Metabolic syndrome (e.g. HTN, T2D, obesity), frequent in cardiometabolic HFpEF phenogroups, also characterized by CKD, have emerged as high-risk in several studies, notably in the US [71]. Moreover, in patients with acute HFpEF, the risk of hospitalization increases cumulatively in proportion to the comorbidity burden [72]. Thus, understanding the immune mechanisms characteristic of distinct HFpEF phenogroups may provide new diagnostic, prognostic, or therapeutic tools to prevent or limit HFpEF development.

### Hypertension

HTN is a well-known driver and a common risk factor of HFpEF and is present in > 50% of patients [68, 73]. HTN promotes a chronic pro-inflammatory and oxidative state that contributes to vascular and myocardial dysfunction. Chronic pressure-overload triggers endothelial activation, initiating microvascular inflammation [6, 74, 75]. Activation of the renin-angiotensin-aldosterone (RAAS) and sympathetic nervous systems further enhances NF-κB and NLRP3 signaling, promoting the release of IL-6, IL-1β, TNF-α, and CCL2/MCP-1, and the upregulation of endothelial adhesion molecules, including ICAM-1 and VCAM-1 [74–76]. Indeed, elevated levels of circulating IL-6, TNF-α, IL-8, and CCL2 were detected in a cross-sectional study of 275 stable hypertensive patients with and without HFpEF [75].

An observational study showed that increased systolic blood pressure is associated with increased number of pro-inflammatory immune cells (NK cells, γδ T cells, and non-classical monocytes) [77]. In addition, in the presence of systemic inflammation, indicated by increased plasma CRP, the risk for development of HTN is increased [75, 78].

HTN is more prevalent with increasing age [79] and this risk clustered with presence of obesity. Increased fat mass influences blood pressure via increased renal sympathetic nervous tone, activating RAAS causing sodium retention in the kidney and systemic vasoconstriction [80]. Interestingly, obesity has a higher correlation with HFpEF compared to HTN, but obesity impacts blood pressure thus confounding the association.

### Diabetes

T2D is more common in HFpEF, where it is associated with disease progression, while type 1 diabetes is more common in HFrEF [33, 81]. T2D, characterized by insulin resistance and a hyperglycemic state, leads to elevated reactive oxygen species (ROS) and lower nitric oxide (NO˙) bioavailability.

Indeed, lower plasma NO˙ levels have been found in HFpEF patients with T2D [82], while IL-6 and IL-8 levels were increased [33, 82]. Moreover, increased NFκB pathway activity, resulting in increased expression of pro-inflammatory mediators (e.g. IL6, TNF-α, ICAM1, VCAM1, and NLRP3), has been found in diabetic HFpEF patients. However, only plasma ICAM-1 levels were significantly increased in these patients [33].

### Obesity

Obesity has been proposed to contribute to HFpEF through metabolic stress-induced inflammation, termed “meta-inflammation” [8], indeed obese patients have increased circulating levels of several inflammatory biomarkers. For example, CRP, serum amyloid A (SAA), and IL-6 were all significantly higher, whereas VCAM-1 and ICAM-1 levels were lower in obese HFpEF patients [2, 7]. Elevated CRP in HFpEF patients with obesity was associated with more severe metabolic derangements [66]. Furthermore, plasma IL-6 levels correlated with levels of NT-proBNP, CRP, and TNF-α in these patients [19]. Of note, obese HFpEF patients commonly also suffer from renal dysfunction, and T2D, and obese patients with T2D have elevated odds of HFpEF [7].

As a high proportion of HFpEF patients are overweight or obese [83], expansion of visceral adipose tissue (VAT) and epicardial adipose tissue (EAT) may contribute to systemic and myocardial inflammation through activation of immune pathways [8]. For example, accumulation of pro-inflammatory lipids drives VAT inflammation, resulting in secretion of inflammatory mediators. EAT expansion in HFpEF patients, linked to increased cytokine production (e.g., TNF-α, IL-1β, IL-6) and immune cell accumulation near the heart, has been linked to worse hemodynamics, reduced exercise capacity, and increased mortality [8]. This highlights the relevance of immune-adipose-myocardial interactions in development or progression of HFpEF.

### Chronic Kidney Disease

CKD is a common comorbidity in both HFpEF and HFrEF, although more prevalent in HFpEF [84]. Importantly, CKD is associated with worse outcome in both HFpEF and HFrEF [4]. The mortality rates of HFpEF patients with CKD is higher than in non-CKD HFpEF [85]. Inflammation-related circulating biomarkers differentiating HFpEF patients with and without CKD have been identified. For example, TNF-α, but not IL-6, levels were significantly higher in a cluster of HFpEF patients with frequent CKD (71,4%) compared to non-CKD patients [86]. In a pig model of CKD, the diseased kidney was shown to be a major source of TNF-α and IL-6 [87]. Age may also influence CKD-induced inflammation, as circulating TNF-α, IL-6, and sST2 levels were found to be elevated in a younger HFpEF phenogroup, whereas NT-pro-BNP was higher in an older CKD phenogroup [71]. In a study of incident HF development in CKD patients, patients developed HFrEF and HFpEF at the same rates, however higher IL-6 and higher GDF-15 were more strongly associated with HFpEF than with HFrEF [88].

#### Atrial Fibrillation

AF is commonly observed in HFpEF patients, notably in aged women [89]. Its pathophysiology includes inflammation, which may share certain features or triggers with systemic and myocardial inflammation in HFpEF [90]. For example, polymorphisms in genes encoding IL-1, IL-6, and IL-10 have been independently associated with AF in humans. However, in a large study, IL-6 was the only circulating cytokine associated with AF [91]. The presence of AF in HFpEF leads to poorer prognosis [92] and worse outcome [93].

Similarly, a study in obesity-associated HFpEF patients found that those with AF had more advanced disease [93]. HF patients (both HFpEF and HFrEF) with AF reportedly had higher plasma levels of IL-6, NT-proBNP, and CRP, coupled to lower eGFR [94]. Further, the subset of HFpEF patients with AF had higher levels of IL-6, were older, and suffered from T2D, and anemia.

### Age and Gender

Age and gender influence the prevalence and severity of HFpEF, and both factors influence immune responses [95]. Indeed, low-grade inflammation accompanies the ageing process and has been termed “inflammaging” [35], and may in part be driven by ageing-associated clonal hematopoiesis of indeterminate potential [96]. Of note, a majority of HFpEF patients are women above 65 years. Due to the metabolic impact of menopause, older women are at increased risk to develop obesity as compared to men, and women with obesity are at greater risk of developing HFpEF [81, 97]. A retrospective analysis of three clinical trials (CHARM-preserved, I-PRESERVE, and TOPCAT) addressed the distribution of age and gender in HFpEF patients: While younger patients tended to be male, with major comorbidities being obesity and T2D, women with HFpEF were on average older, with higher prevalence of AF, HTN, and CKD [79]. Similar results have been reported in other studies [68, 89, 98]. Thus, age and gender may influence immune responses both *directly* (inflammaging and hormone-mediated immune effects), and *indirectly*, through their impact on development of HFpEF-associated co-morbidities, out of which obesity and/or CKD appear to be associated with especially poor prognosis.

### Current and Emerging Therapeutic Strategies

Although low-grade inflammation and immune dysregulation have long been proposed to play a central role in the pathophysiology of HFpEF, the translation of these insights into effective therapies remains challenging. One important challenge is the fact that cardiac inflammation cannot be measured directly. Echocardiographic parameters capture the functional and structural effects of inflammation-driven myocardial remodeling, manifesting as impaired relaxation, increased filling pressures, and left atrial and ventricular enlargement [99]. Cardiac magnetic resonance imaging (MRI) allows for characterization of tissue characteristics. Late gadolinium enhancement detects focal fibrosis, while T1 mapping and extracellular volume quantification evaluates the expansion of the extracellular component and can estimate diffuse fibrosis. Furthermore, T2 mapping quantifies myocardial oedema as an indirect measure of inflammation [100]. Echocardiography and MRI provide complementary assessments of structural and functional myocardial abnormalities associated with inflammation and fibrosis; however, both modalities remain indirect measures and do not allow direct visualization or quantification of active myocardial inflammation. Another challenge is the heterogeneity. Most pharmacologic interventions in HFrEF have failed to produce beneficial effects in HFpEF, likely due to its pathophysiological heterogeneity [101, 102]. However, recent advances have yielded new clinical treatment strategies for HFpEF.

### Current Therapies for HFpEF

#### SGLT2 Inhibitors

Sodium-glucose cotransporter 2 (SGLT2) inhibitors, including empagliflozin and dapagliflozin, have shown benefits in HFpEF. In both the EMPEROR-Preserved and DELIVER trials, SGLT2 inhibition reduced DHFA risk by 20% in ≈ 6000 HF patients with a LVEF > 40%. These benefits were mainly due to decreased HF hospitalization [103, 104]. The kidney proximal tubules are the main targets of SGLT2 inhibition, where it leads to reduction of sodium and glucose reabsorption, diminishing glomerular pressure and hyperfiltration [105–107].

However, besides controlling blood glucose levels, SGLT2 inhibitors exert pleiotropic effects relevant for HFpEF pathophysiology [108]. For example, they suppress NLRP3 inflammasome activation, thereby attenuating the release of pro-inflammatory cytokines, including IL-1β and IL-18 [109–113]. Additionally, SGLT2 inhibitors have been found to exert anti-inflammatory effects in pro-inflammatory macrophage, by attenuating NF-κB, MAPK, and JAK/STAT pathways, thereby decreasing TNF-α and IL-6 production, while promoting alternative macrophage polarization [109–111]. SGLT2 inhibition may also improve vascular endothelial function through increased NO˙ signaling and decreased arterial stiffness [114, 115].

Empagliflozin attenuates microvascular dysfunction by reducing endothelin-1 activity, promoting the activation of beneficial PI3K/Akt/eNOS pathway and enhanced insulin-induced vasodilatation [116]. Additionally, SGLT2 inhibitors promote ketogenesis, a metabolic pathway that generates ketone bodies, which may improve cardiac energy efficiency [117].

#### MRAs

Mineralocorticoid receptor antagonists (MRAs), including spironolactone and eplerenone, are commonly used to manage HF. They mitigate the actions of aldosterone, leading to reduced myocardial fibrosis, vascular inflammation, and sodium retention, all processes tightly linked to HFpEF pathophysiology. However, the randomized controlled TOPCAT trial, which evaluated MRA treatment with spironolactone including in > 3000 patients with LVEF ≥ 45%, did not meet its primary endpoint of prevention of cardiovascular death, cardiac arrest, or HF hospitalization. However, pre-specified regional analyses revealed substantial heterogeneity in study participants. Promisingly, in American HFpEF patients, spironolactone significantly reduced the primary endpoint by 17%, notably by decreasing HF hospitalizations, whereas no benefit was observed in Russian/Georgian patients [118]. Moreover, clinical data have demonstrated anti-inflammatory effects of MRAs through reduction of circulating TNF-α, IL-6, IFN-γ, sST2, and galectin-3 levels [119, 120]. However, evidence regarding more conventional inflammatory markers, including CRP, remains inconsistent [121].

More recently, finerenone, a selective non-steroidal MRA, was developed [122, 123]. Two large phase III trials, FIDELIO-DKD and FIGARO-DKD, reported reductions in renal failure, cardiovascular events, and mortality in patients with T2D and diabetic CKD [124, 125]. These effects were associated with suppression of pro-inflammatory cytokines, attenuation of oxidative stress, and inhibition of fibrosis-related pathways. Evidence for finerenone in HFpEF is directly supported by the FINEARTS trial, demonstrating a 16% reduction in the primary endpoint of worsening HF events [126]. Its anti-inflammatory and anti-fibrotic properties, together with favorable renal effects, suggest potential advantages of finerenone over steroidal MRAs [122, 123].

#### ARNIs

Combined angiotensin II type 1 receptor and neprilysin inhibitors (ARNIs), including sacubitril/valsartan, act by enhancing ANP and BNP signaling while attenuating RAAS activation. This dual mechanism promotes natriuresis, vasodilatation, and anti-fibrotic activity, all of which are relevant to HFpEF pathophysiology. The PARAGON-HF trial evaluated sacubitril/valsartan including in patients with LVEF ≥ 45%. Although the trial did not achieve significant results for its primary endpoint (HF hospitalizations and cardiovascular death), exploratory analyses suggested benefit in subgroups, particularly in women and patients with LVEF 45–57%. These findings suggest that ARNI therapy may be effective in specific HFpEF phenogroups [127, 128]. ARNIs may also modulate inflammation. Indeed, both ANP and BNP suppress pro-inflammatory signaling pathways and reduce oxidative stress [129]. Clinical studies have shown reductions in circulating CRP, as well as fibrosis biomarkers, such as procollagen type I C-terminal propeptide (PICP), following sacubitril/valsartan therapy, indicating their ability to attenuate systemic inflammation and ECM remodeling [130–132] .

### HFpEF Phenogroup-selective Effects of Treatment?

As mentioned above, the multifactorial etiology of HFpEF, which includes advanced age and female sex together with several comorbidities [71], contributes to its heterogenous clinical presentation, constituting a particular challenge for development of therapies. Some of the HF treatments above may show benefit in HFpEF by limiting the load of co-morbidities. For example, the EMPA-REG OUTCOME trial showed, in patients with T2DM at high cardiovascular risk, that empagliflozin reduced cardiovascular death and HF hospitalization by 35%, while also decreasing body weight and blood pressure [133]. Similarly, GLP-1 receptor agonists hold promise in the treatment of obesity-related HFpEF. Indeed, the STEP-HFpEF trial demonstrated that semaglutide improved HF-related symptoms, body weight, and reduced inflammatory biomarkers, including CRP and NT-proBNP [134]. Likewise, in the SUMMIT trial, tirzepatide reduced the combined risk of cardiovascular death or worsening HF, while also improving blood pressure, estimated plasma volume, body weight, and systemic inflammation [135]. Further studies are needed to elucidate whether GLP-1 receptor agonists display beneficial effects, beyond weight loss, on inflammatory parameters in HFpEF.

In addition to metabolic disorders, both HTN and systemic inflammation promote cardiac remodeling and (diastolic) dysfunction, notably by driving myocardial fibrosis. For these patients, MRAs may be highly relevant as they target aldosterone-driven fibrosis. In a TOPCAT subanalysis, only the subgroup characterized by CKD, obesity, T2D, and myocardial fibrosis showed a 25% risk reduction in the primary outcome with spironolactone. Notably, finerenone may have stronger anti-fibrotic and anti-inflammatory effects through its selective receptor binding, suggesting a stronger therapeutic potential compared to steroidal MRAs [122, 123]. Nevertheless, adequate clinical trials are needed to prove this point.

Renal impairment with systemic inflammation is a characteristic of another common HFpEF phenogroup with poor prognosis. In these patients, SGLT2 inhibitors reduced the risk of renal outcomes [107, 136]. Likewise, finerenone treatment reduced the risk of kidney failure, renal mortality, and CKD progression through its anti-inflammatory and anti-fibrotic properties [124]. As patients with renal dysfunction frequently have high NT-pro-BNP levels, ARNIs may offer additional benefit by enhancing NP signaling and improving cardiorenal hemodynamics [129, 132].

Together, these findings indicate that current or emerging therapies for HFpEF not only mitigate cardiovascular outcomes, but also modify key comorbidities, including obesity, T2D, HTN, and CKD, thereby addressing specific drivers of HFpEF progression. A personalized approach focused on comorbidities and their underlying pathophysiology in specific HFpEF phenogroups is likely necessary to demonstrate benefit of targeted interventions.

### Inflammation-targeted Therapies

As chronic low-grade inflammation plays a prominent role in the pathophysiology of HFpEF, anti-inflammatory therapies are gaining attention. Although current pharmacological treatment options, as mentioned above, have shown some anti-inflammatory effects, recent trials are now exploring biotherapy approaches that directly target inflammation in HFpEF.

#### IL-6 Blockade

Elevated IL-6 levels are frequently observed in HFpEF and have been associated with disease severity and mortality. In the setting of myocardial infarction, anti-IL-6R-targeted therapies, such as tocilizumab, have shown systemic anti-inflammatory effects [137]. Furthermore, the RESCUE trial reported that a human monoclonal anti-IL-6 antibody, ziltivekimab, reduced inflammatory biomarkers, including CRP and neutrophil-lymphocyte ratio, in high-cardiovascular-risk CKD patients [138]. These findings support the therapeutic potential of IL-6 blockade in HFpEF-related inflammation.

The ongoing HERMES trial (https://clinicaltrials.gov/study/NCT05636176), is currently investigating whether ziltivekimab may improve clinical outcomes in specific HFpEF patients with CRP ≥ 2 mg/L and elevated NT-pro-BNP [139].

#### IL-1 Blockade

The CANTOS trial provided evidence that anti-IL-1β treatment, with canakinumab, reduces systemic inflammation and cardiovascular events in patients with prior myocardial infarction and CRP ≥ 2 mg/L [140]. In HFpEF, the D-HART pilot study examined the effects of IL-1 blockade, with anakinra (recombinant soluble IL1RA), in 12 patients with plasma CRP > 2 mg/L. Treatment for 14 days significantly improved VO_2_max and reduced CRP levels, providing preliminary evidence of improved aerobic exercise capacity and reduced systemic inflammation [141]. However, in the larger D-HART2 trial, evaluating 12 weeks of anakinra in a similar population, the primary endpoint (change in VO_2_max) was not reached, although CRP and NT-pro-BNP levels were decreased. The potential impact of anti-IL-1β therapy on cardiac remodeling and dysfunction in HFpEF requires further studies.

#### NLRP3 Inflammasome Modulation

Colchicine, an anti-inflammatory drug, inhibits activation of the NLRP3 inflammasome, thus reducing IL-1β activation. Its efficacy in cardiovascular diseases has been demonstrated in the LoDoCo and COLCOT trials, reporting reduction of adverse events in patients with chronic coronary artery disease [142] and after myocardial infarction [143]. Currently, its therapeutic potential in HFpEF is being investigated in several trials. The COLHEART-PRESERVED (https://clinicaltrials.gov/study/NCT06081049) and LoDoCo-HFpEF (https://www.clinicaltrials.gov/study/NCT06130059) studies are evaluating the quality of life, functional capacity, and cardiac function following colchicine treatment. The COLpEF trial is focusing specifically on its effect on inflammatory biomarkers, including CRP and sST2, in symptomatic HFpEF patients [144]. While data on clinical outcomes are not available yet, its anti-inflammatory profile makes it an attractive candidate. However, the specificity of colchicine for NLRP3 remains debated, therefore, more selective agents may be needed to fully target this pathway in HF [145, 146].

#### Myeloperoxidase Inhibition

Myeloperoxidase (MPO), a neutrophil-derived enzyme, is another emerging target linking systemic inflammation to the development of HFpEF. MPO is known to cause oxidative stress, microvascular dysfunction, myocardial fibrosis, and diastolic dysfunction. A selective MPO inhibitor, mitiperstat (AZD4831), is currently under investigation in HFpEF. The phase IIa SATELLITE trial [141] demonstrated a good safety profile and confirmed its ability to inhibit MPO. However, Popovic et al. reported no improvements in cardiac or pulmonary vascular hemodynamics after acute MPO inhibition with mitiperstat [147, 148]. Consistently, the ENDEAVOR trial [142] failed to demonstrate that 16 weeks of mitiperstat treatment could improve outcome or exercise capacity in HFpEF [142, 143]. These neutral findings suggest that established myocardial stiffness and vascular remodeling is irreversible and given the multifaceted nature of oxidative stress, suppression of MPO may disrupt physiological redox signaling rather than confer benefit [148]. Together, these findings indicate that MPO inhibition may have limited therapeutic value in patients with HFpEF.

## Conclusions

Low-grade inflammation is a hallmark of HFpEF, yet mounting evidence shows that age, gender, and diverse comorbidities shape the immune landscape driving disease onset and progression. Fibrosis, another cornerstone of HFpEF pathophysiology, is tightly interwoven with inflammation, underscoring the need for therapies that target both processes. Beyond anti-inflammatory therapies, anti-fibrotic strategies, including emerging treatments, e.g. pirfenidone [149], are promising adjuncts.

Importantly, the clinical heterogeneity of HFpEF has pushed the research community towards development of phenogrouping approaches, including unsupervised machine-learning, to integrate multiple clinical and biochemical variables to refine risk stratification. As precision medicine advances, a deeper understanding of comorbidity-driven immune remodeling, including “meta-inflammation” and “inflammaging” (Fig. 1), may pave the way for tailored treatment combinations. Such strategies hold the potential to transform HFpEF care from generalized management to mechanism-based interventions, thus ushering in the paradigm shift this patient population urgently needs.

##  Key References

- Paulus WJ, Tschöpe C. A novel paradigm for heart failure with preserved ejection fraction: Comorbidities drive myocardial dysfunction and remodeling through coronary microvascular endothelial inflammation. J Am Coll Cardiol. 2013;62:263–71.
  - **○ **Manuscript describes the involvement of inflammation in HFpEF.
- Ramirez MF, Lau ES, Parekh JK, Pan AS, Owunna N, Wang D, et al. Obesity-Related Biomarkers Are Associated With Exercise Intolerance and HFpEF. Circ Heart Fail. 2023;16:E010618.
  - **○ **Paper demonstrates the involvement of obesity in HFpEF.
- Cohen JB, Schrauben SJ, Zhao L, Basso MD, Cvijic ME, Li Z, et al. Clinical Phenogroups in Heart Failure With Preserved Ejection Fraction: Detailed Phenotypes, Prognosis, and Response to Spironolactone. JACC Heart Fail. 2020;8:172–84.
  - **○ **Randomized clinical trial establishing phenogroups in HFpEF.

## Data Availability

No datasets were generated or analysed during the current study.
